# Guide to extraluminal fish bone retrieval with serial computed tomography scans: a case series

**DOI:** 10.1186/s13256-024-04719-5

**Published:** 2024-08-15

**Authors:** Ting Ting Yew, Ing Ping Tang, Li Yun Lim, Yuanzhi Cheah, Shiong Leong Yew

**Affiliations:** 1 Department of Radiology, Faculty Medicine and Health Sciences, Universiti Malaysia Sarawak, Kota Samarahan, Sarawak Malaysia; 2 Institute of Borneo Studies, Kota Samarahan, Sarawak Malaysia; 3 Department of Otorhinolarynology Head & Neck, Faculty Medicine And Health Sciences, Universiti Malaysia Sarawak, Kota Samarahan, Sarawak Malaysia; 4 Department of Otorhinolarynology Head & Neck, Sarawak General Hospital, Kuching, Sarawak Malaysia; 5 Department of Radiology, Sarawak General Hospital, Kuching, Sarawak Malaysia

**Keywords:** Case report, Fish bone, Foreign body migration, Serial computer tomography, Multiplanar reconstruction

## Abstract

**Background:**

Fish bone ingestion is commonly encountered in emergency department. It poses a diagnostic and therapeutic challenge particularly when it migrates extraluminally, necessitating a comprehensive and multidisciplinary approach for successful management.

**Case presentation:**

Here we reported four cases of extraluminal fish bone. The first patient was a 68-year-old Chinese man who had odynophagia shortly after a meal involving fish. The second was a 50-year-old Iban man who reported a sharp throat pain after consuming fish 1 day prior. The third patient was a 55-year-old Malay woman who developed throat pain and odynophagia after consuming fish 1 day earlier. The fourth patient, a 70 year-old Iban man, presented late with odynophagia, neck pain, swelling, and fever 1 week after fish bone ingestion. These unintentional fish bone ingestions faced challenges and required repeat computed tomography scans using multiplanar reconstruction in guiding the surgical removal of the fish bone.

**Conclusion:**

We underscore the significance of multiplanar reconstruction in pinpointing the fish bone’s location, demonstrating the migratory route, and devising an accurate surgical plan.

## Background

Unintentional fish bone (FB) ingestion is a common clinical scenario that presents a unique set of challenges for both patients and healthcare providers. FB commonly lodges in various area of the upper aerodigestive tract, including the tonsils, tongue base, vallecula, piriform fossa, or upper esophagus [1]. Nevertheless, some sharp and pointed FB might locate extraluminally and migrate into the soft tissue and deep space of the neck. This may result in severe complications such as perforation, abscess formation, and puncture of major vessels resulting in bleeding [2]. The radiological evaluation of such cases, particularly with the use of serial computed tomography (CT) scans, plays a pivotal role in guiding therapeutic interventions and ensuring optimal patient outcomes.

We present cases of four patients with FB that migrated to oropharyngeal mucosal, parapharyngeal, and prevertebral spaces, which required serial CTs for successful removal (Table 1). Our cases highlights the significance of employing multiplanar reconstruction (MPR) to pinpoint the location of FB and develop a detailed surgical strategy.

**Table 1:** Summary of the four cases

Patient	Age/sex	Duration of symptoms	FB location	Complication	Approach
1	68/male	< 1 day	Hypopharynx	Migrated to cervical esophagus	External transcervical
2	50/male	1 day	Right pharyngeal mucosa space	Nil	Tonsillectomy and lateral neck exploration
3	55/female	1 day	Right prevertebral space	Broken into segments and pneumomediastinum	Intraoral exploration
4	70/male	7 days	Right parapharyngeal space	Neck abscess	External incision and drainage, then lateral neck exploration

## Case reports

### Case 1

A 68-year-old Chinese male patient presented with complaint of odynophagia shortly after a meal involving fish consumption on the same day. Oropharyngeal examination and endoscopic laryngeal examination did not reveal the FB.

**Fig. 1:**
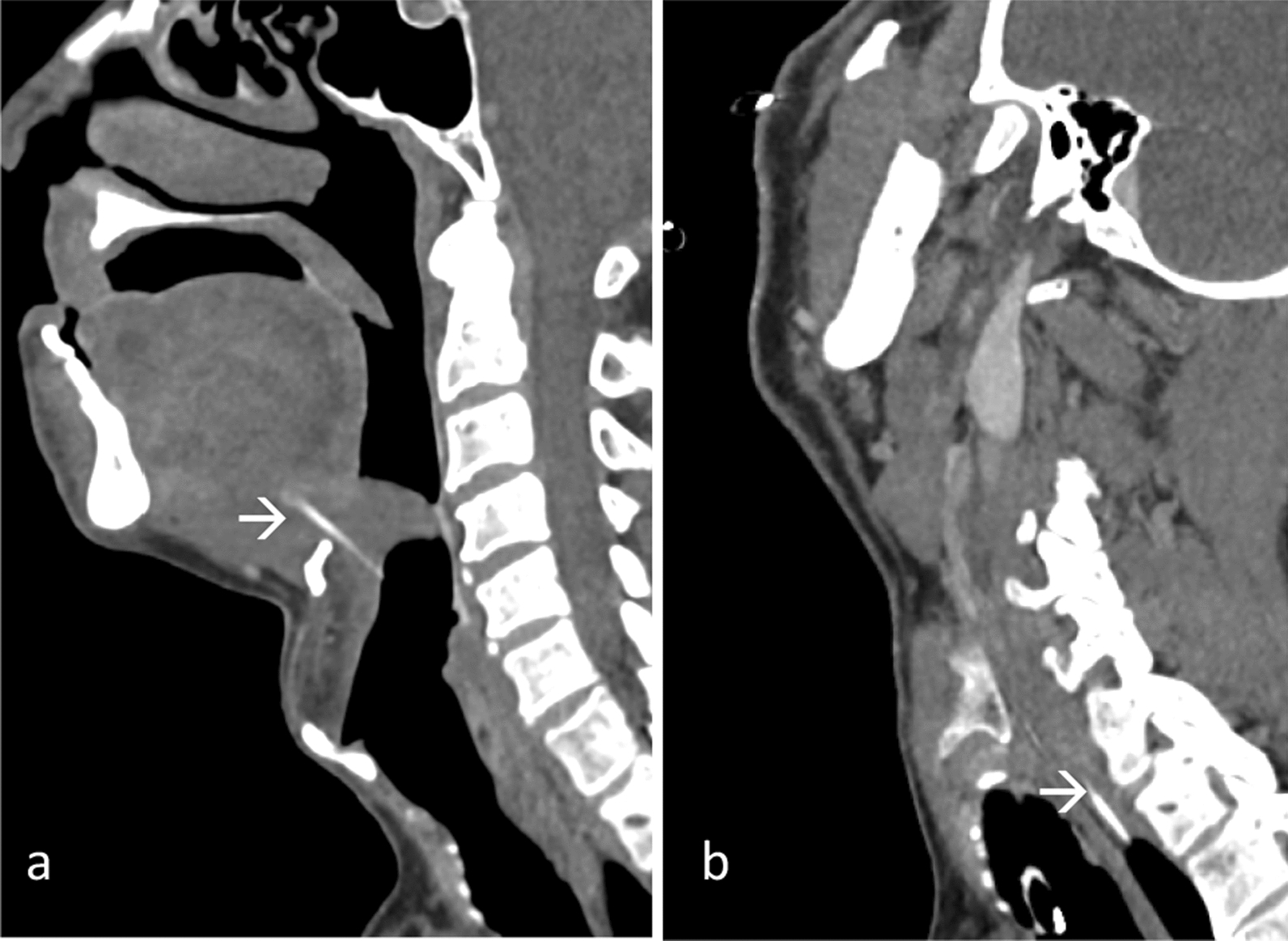
Contrast-enhanced sagittal multiplanar reconstruction images **a** showed a linear fish bone (arrow) at tongue base of hypopharynx region, **b** which later migrated to lateral wall of cervical esophagus (arrow)

Lateral neck radiograph showed a thin vertically orientated FB at the suprahyoid region anterosuperior to the epiglottis. A CT neck with intravenous contrast identified the FB at the base of tongue of the hypopharynx region (Fig. 1a). During the emergency examination under anesthesia (EUA) and direct laryngoscopy (DL), a hematoma was present at the left vallecular region with a punctum on the left laryngeal surface of epiglottis; despite this, the FB was not found.

A subsequent CT neck with intravenous contrast performed 3 days later indicated migration of the FB to right lateral wall of the cervical esophagus (Fig. 1b). During the neck exploration, DL, esophagoscopy, a 3.1 cm FB was identified in the soft tissue between the esophagus, posterior to the right carotid, and anterior to the pleura, and was successfully removed via external transcervical method. Additionally, direct laryngoscopy revealed a fractured piece of the FB, which was removed from the right vallecular region. It was uneventful postoperatively.

### Case 2

A 50-year-old Iban male patient reported a sharp pain in his throat after consuming fish 1 day prior. Oropharynx examination revealed redness on the right soft palate and a small laceration wound at the right vallecular, but the FB was not visible.

**Fig. 2:**
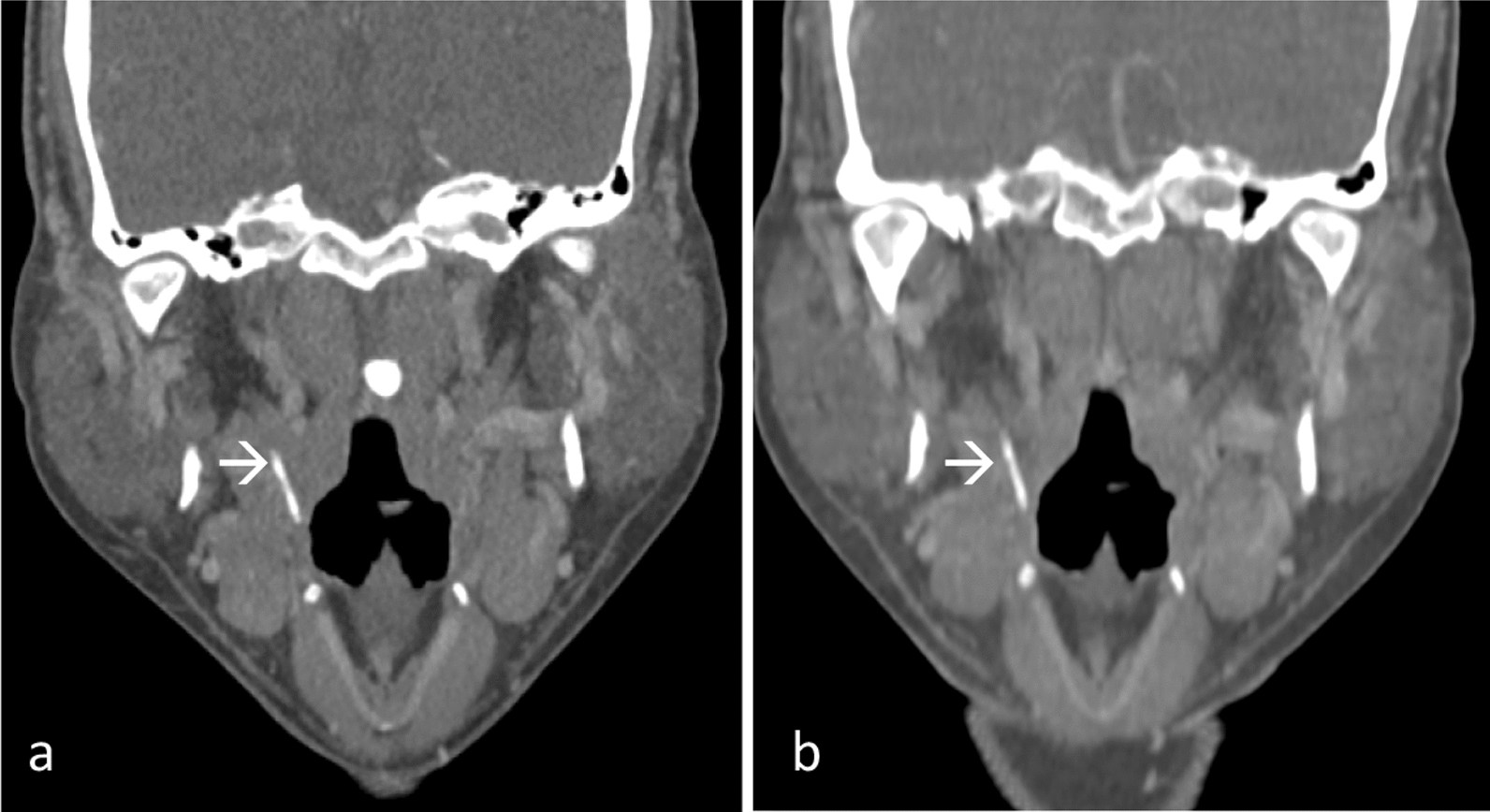
Contrast-enhanced coronal multiplanar reconstruction images **a** showed a linear fish bone (arrow) at right pharyngeal mucosa. **b** Repeat scan showed fish bone at the similar location (arrow)

A CT neck with intravenous contrast disclosed the linear FB embedded in the right pharyngeal mucosa (Fig. 2a). EUA and DL were conducted but the FB could not be located despite a careful search. The patient was given antibiotics for several days. Upon follow-up, the patient was symptom free. Flexible scope revealed slough at the right pharyngoepiglottic fold. A second CT neck with intravenous contrast showed the FB still within the right pharyngeal mucosa space (Fig. 2b).

A second EUA and DL was performed, however, the FB remained elusive, leading to a decision to perform a right tonsillectomy. The tonsillar bed was dissected to explore the right parapharyngeal space. Eventually, the 1.7 cm FB was identified encased by soft tissue in the right parapharyngeal space, superior to the right submandibular gland. Postoperatively, the patient recovered well.

### Case 3

A 55-year-old Malay female patient experienced throat pain and odynophagia after consuming fish 1 day earlier. A 70-degree endoscopy examination revealed a FB in the left pyriform fossa, accompanied by swelling in the left arytenoid. Attempts to remove the FB were unsuccessful.

**Fig. 3:**
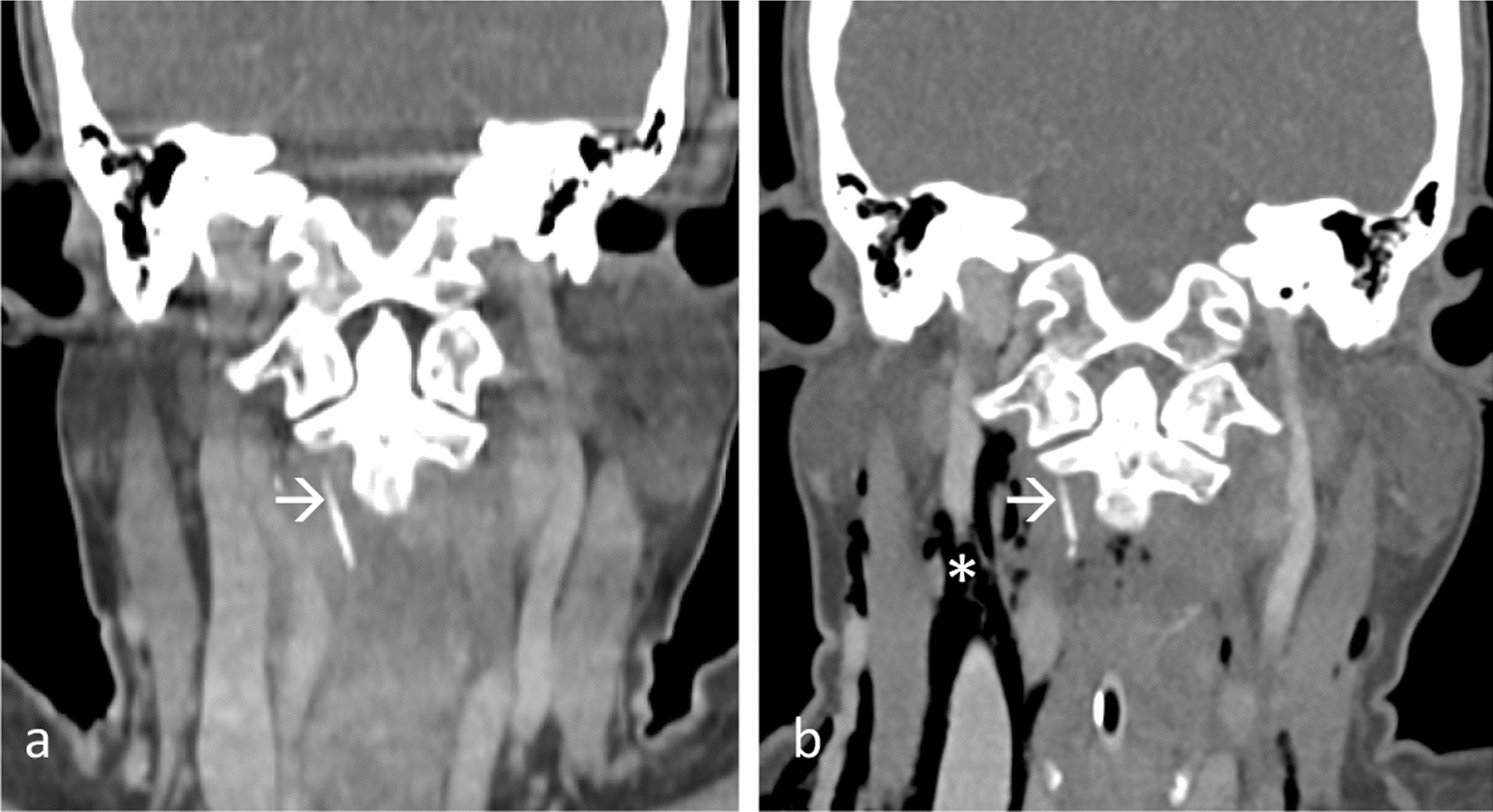
Contrast-enhanced coronal multiplanar reconstruction images **a** showed a linear fish bone (arrow) at right prevertebral region. Repeat CT **b** revealed the fish bone (arrow) at the similar region but broken into segments, complicated with pneumomediastinum (asterisk)

EUA, DL, and esophagoscopy were conducted. A small ulceration was spotted at the post-cricoid region, but no foreign body was detected. A CT neck with intravenous contrast disclosed a linear FB in the right prevertebral mucosal space of the oropharynx at the C2/3 level (Fig. 3a).

A second DL and esophagoscopy were carried out, involving an incision over the posterior oropharyngeal wall extending to the prevertebral muscle. Despite efforts, the FB could not be located. Second CT neck indicated the FB still situated in the same location but with pneumomediastinum (Fig. 3a).

In third DL with intraoral exploration, the 2.4 cm FB was discovered broken into four segments, embedding in the right prevertebral muscle in a lateral oblique direction.

Following the operation, the patient received antibiotics for 1 week and follow-up revealed well-healed intraoral wounds.

### Case 4

A 70-year-old Iban male patient, with poorly-controlled diabetes mellitus, experienced odynophagia, neck pain with swelling, and fever for 1 week after a FB lodged in the floor of his mouth. He has trismus and muffled voice, with raised floor of the mouth on oral cavity examination. A flexible scope identified edematous epiglottis and tongue base.

**Fig. 4:**
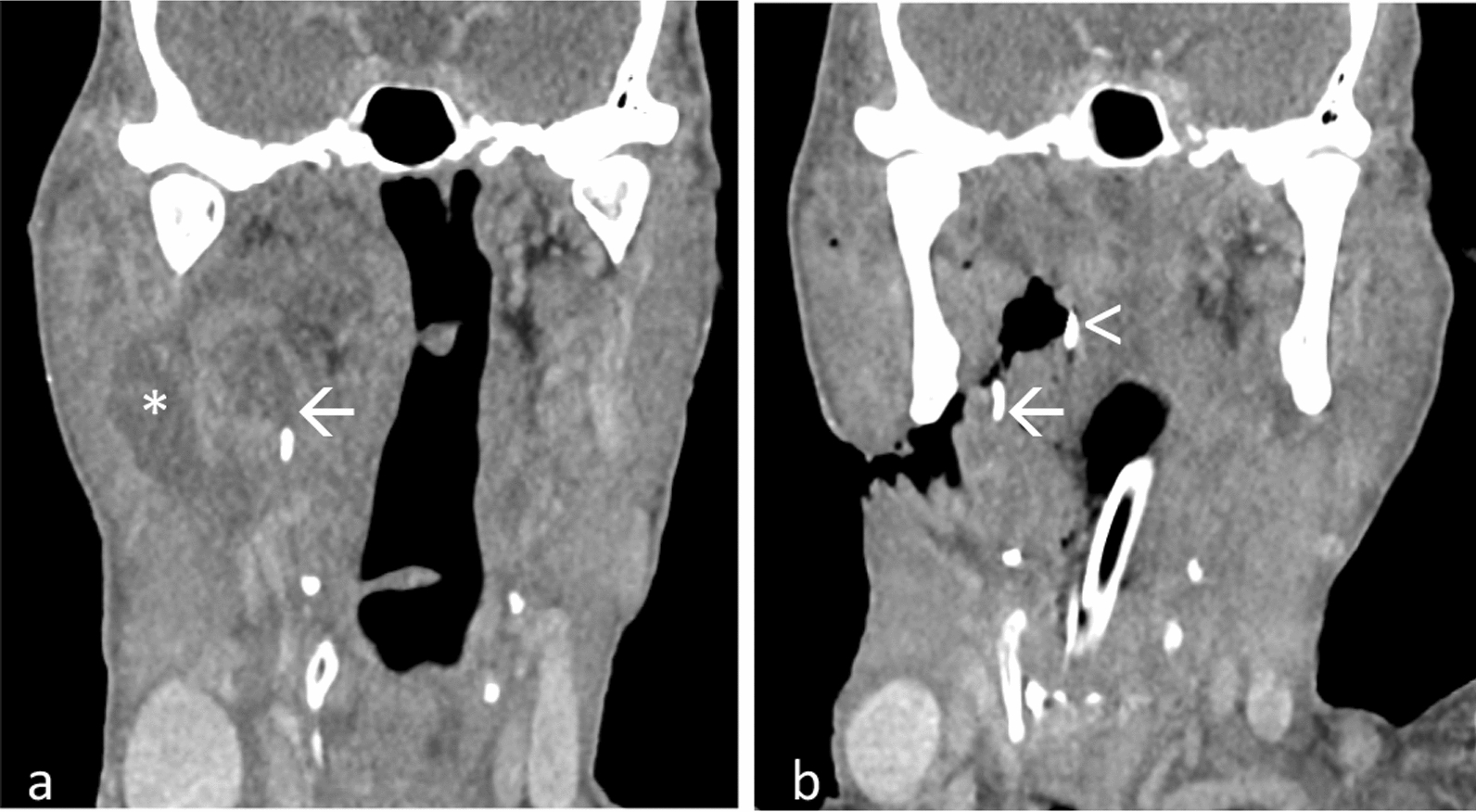
Contrast-enhanced coronal multiplanar reconstruction images **a** showed a linear fish bone (arrow) with abscess (asterisk) at right submandibular region. **b** Repeat computed tomography showed the fish bone (arrow) migrated to parapharyngeal region, lateral to the surgical patty (arrow head)

A CT neck with intravenous contrast revealed a linear FB in the right submandibular region, along with a multi-loculated rim-enhancing collection at the floor of the mouth (Fig. 4a). Intravenous antibiotics were administered empirically. An external incision and drainage revealed an abscess cavity extending from the right submandibular to the left submandibular space with 80 ml of pus drained, but no FB was found.

A repeat CT neck identified the FB located at the right parapharyngeal space. Subsequent neck exploration noted a defect in the right lateral pharyngeal wall and right anterior pillar. The FB could not be identified despite a thorough exploration. Intraoperatively, X-ray image intensifier (II) and a surgical patty placed in the right parapharyngeal space for location assistance proved unsuccessful.

A subsequent CT scan, with the surgical patty in place, revealed the FB had penetrated to the right parapharyngeal region, lateral to the surgical patty (Fig. 4b). Immediate re-exploration of the neck wound revealed a 1.5 cm FB at the right lateral pharyngeal wall at the tongue base level. Postoperatively, the antibiotic regimen was upgraded to IV ceftriaxone, and insulin was initiated to controlled the blood glucose. Another wound debridement, secondary suturing, and reconstruction with a pectoralis major rotational flap were performed 2 weeks later. Patient was discharged home after 3 weeks of hospital care.

## Discussion

When the FB is situated in the upper aerodigestive tract, a lateral neck radiograph is typically the initial ordered radiological examination, as 90% of FB (90%) are lodged in the suprahyoid location [3]. Nonetheless, plain radiography demonstrates a low sensitivity (only 32%) for detecting FB in the upper aerodigestive tract and esophagus [4], especially in specific scenarios when the FB is lodged at the tonsillar level or the base of the tongue, which means it may not be visible on radiograph. Various studies prove radiography to be an unreliable method for diagnosing FB ingestion [5, 6], as the radiopacity of the bone varies depending on the fish species [7, 8]. Even when FB exhibits sufficient radiopacity for visualization on radiographs, the presence of soft tissue and fluid can obscure the minimal calcium content of the fish bone, especially in obese individuals [5]. In our center, plain radiographs is performed for detecting FB that impacted within the cervical esophagus or bronchus [9]. Other than linear calcific structure of FB, additional information that can be elicited on a lateral radiograph include the widening of prevertebral soft tissue and the presence of retropharyngeal air.

CT neck is the preferred and the most sensitive imaging for precise localization of the FB [11, 12], especially when there is definite history of FB embedding and no FB visible on oral cavity and oropharyngeal examination. CT is particularly important to assess the location of the FB and determining its depth from adjacent bony landmark prior to EUA. The anatomical relationship between the extraluminal FB and vital structures in the neck, such as the trachea, esophagus, cervical vertebrae, thyroid gland, and neck arteries, can be precisely delineated. Moreover, CT assists in better delineating associated complications, such as perforation or abscess formation. CT scans are also valuable in identifying the migration of the FB to a location outside the aerodigestive tract [12].

In our center, patients with FB ingestion typically undergo CT scan of neck with intravenous contrast medium and the scan obtained in the portal venous phase. FB typically appears as a calcified linear structure and is usually enveloped by surrounding inflammatory tissue. Challenges arise when the FB migrates extraluminally, as it may resemble a blood vessel, particularly following the administration of intravenous contrast [6, 13], leading to potential oversight. This can be discerned with careful windowing of the CT images, as FB generally exhibits higher attenuation than blood vessel. Thinner CT slices also prove to enable better visualization and differentiation of structures [13]. In our cases, all CT scans were acquired with 1-mm slices, a thickness which is reliable for detecting FB and distinguishing it from a blood vessel.

Positive oral contrast medium was not used in these cases, as it is generally unnecessary for diagnosing most cases according to recent literature reviews [7, 8]. Instead, it might obscure the faint calcification of a fish bone within the lumen of aerodigestive tract, leading to potential under-detection [13].

The comprehensive imaging capabilities of CT, including high resolution, thin collimation, and MPR, facilitate a thorough survey in various projections [5, 13]. Our cases also demonstrated that repeated computed tomography (CT) scans offer a more elucidating depiction of the FB migratory path over time. By employing axial, sagittal, and coronal MPR images, a comprehensive visualization of the FB is facilitated, showcasing its length, location, and direction. Specifically in our cases, the distance of the FB in relation to adjacent soft tissue and bony landmarks such as thyroid cartilage, cricoid, hyoid, and mandible is worth noting to convey in the documentation, as this information is crucial in detecting the precise location and guiding the different optimal approach of the surgeons for FB removal. It is important to consider that the precise location of FB and its distance from nearby landmarks may vary over the time, given that the patient’s neck is in an extended position on the surgical table, as opposed to the neutral neck position on the CT gantry table, thus the actual distance may be elongated. Consequently, serial CT scans are crucial in providing real-time information regarding the FB location and migration, especially when there is negative retrieval despite thorough exploration. In our cases, the FB were extracted via external approach, except for patient 3, where it was extracted with intraoral approach after repeated CTs.

## Conclusion

This series of four reports emphasizes the indispensable contribution of radiologists in the diagnosis and management of ingested fish FB. The utilization of serial CT scans and MPR as a guiding tool not only enhanced the precision of the intervention, but also contributed to a successful outcome for the patient. This also underscores the significance of a collaborative and multidisciplinary approach, where the radiologist’s expertise is integral to the overall care and resolution of complex cases involving FB ingestion.

## Data Availability

Not applicable.

## Abbreviations

FB: Foreign body
CT: Computed tomography
MPR: Multiplanar reconstruction
EUA: Examination under anesthesia
DL: Direct laryngoscopy
